# Fetal abdominal obesity and the ensuing adverse perinatal outcomes in older obese pregnant women with or without obesity and with normal glucose tolerance

**DOI:** 10.1038/s41598-023-43362-w

**Published:** 2023-09-27

**Authors:** Wonjin Kim, Soo Kyung Park, Yoo Lee Kim

**Affiliations:** 1 Division of Endocrinology and Metabolism, Department of Internal Medicine, CHA Gangnam Medical Center, CHA University School of Medicine, 566, Nonhyeon-ro, Gangnam-gu, Seoul, 06135 Republic of Korea; 2 Yonsei University College of Medicine, Seoul, 03722 Republic of Korea; 3 Department of Biostatics and Data Science, University of Texas, Health Science Center at Houston, Houston, TX 77030 USA

**Keywords:** Endocrinology, Medical research

## Abstract

To investigate whether the increased risk of fetal abdominal obesity (FAO) is present in the older (≥ 35 years) and/or obese (≥ body mass index 25 kg/m^2^) women with normal glucose tolerance, we reviewed medical record of 6721 singleton pregnancy. At 24–28 gestational weeks (GW), fetal abdominal overgrowth was assessed by the fetal abdominal overgrowth ratios (FAORs) of the ultrasonographically estimated gestational age (GA) of abdominal circumference per actual GA by the last menstruation period, estimated GA of biparietal diameter or femur length, respectively. FAO was defined as FAOR ≥ 90th percentile. Compared to young and non-obese women, older women showed significantly higher FAORs irrespective of obesity and the prevalence of FAO in older and non-obese women was significantly higher (11.8% vs. 8.6%, *p* < 0.05). The odds ratio for large for gestational age at birth were 3.06(1.96–4.77, *p* < 0.005), 1.47(1.16–1.86, *p* < 0.005) and 2.82(1.64–4.84, *p* < 0.005) in young and obese, older and non-obese, and older and obese women, respectively. The odds ratio for primary cesarean delivery in older and non-obese women was 1.33 (1.18–1.51, *p* < 0.005). An increased risk of FAO at 24–28 GW and subsequent adverse perinatal outcomes have been observed in the older women with or without obesity, compared to younger and non-obese women, despite normal glucose tolerance.

## Introduction

It is well known that fetal growth is the result of interaction between genetic, intrauterine, and maternal factors^1–5^. Recently, a large number of studies have demonstrated relationships between in utero fetal experience and later risk for childhood obesity^2,6,7^ and adult chronic diseases including cardiovascular disease^8–11^. Advanced maternal age and pre-pregnancy obesity were associated with increased risk of adverse perinatal outcomes including gestational diabetes mellitus (GDM)^12–15^, large for gestational age (LGA) and increased fat mass^16–19^, and cesarean section^18,19^. Higher neonatal birth weight was associated with higher body mass index (BMI) leading to obesity^7^ and increased cardiovascular risk profile later in life^20^. Collectively, these findings suggest that considering the antecedents of future disease risk occurring very early in fetal life, developing a new paradigm of interventions starting well before and early in pregnancy would be necessary to tackle the adult-onset disorders such as obesity and cardiovascular disease.

In our previous studies, we observed that fetal abdominal obesity (FAO) was observed as early as 20–24 gestational weeks (GW) in the older women with or without obesity but not in the younger and non-obese GDM subjects^21^, and despite appropriate treatment for GDM, the FAO in the older women with or without obesity detected at the time of GDM diagnosis persisted until delivery with the resultant LGA at birth^12,22^. In the present study, we thus investigated whether the increased risk of FAO was observed in the older women with or without obesity but not in the young and non-obese women with normal glucose tolerance (NGT), and if so, FAO detected at 24–28 GW in the older women with or without obesity persisted until delivery with the ensuing adverse perinatal outcomes such as LGA and increased cesarean delivery.

## Materials/subjects and methods

### Subjects and data collection

We retrospectively reviewed the medical records of 7820 singleton pregnant women who were followed up and delivered at CHA Gangnam Medical Center from January 1, 2012, to April 31, 2015. The data on maternal height and body weight at pre-pregnancy, at the 50-g glucose challenge test (GCT), and near term, biochemical test, and fetal biometry measured on the same day of 50-g GCT, infant birth weight, and mode of delivery were obtained from the medical records. The data collection was approved by the Institutional Review Board of CHA Gangnam Medical Center with a waiver of informed consent for the retrospective chart review (IRB No.GCI-18-10). All experiments were performed in accordance with relevant guidelines and regulations.

### Selection of subjects with NGT after exclusion of GDM and one value abnormality

As described previously^12^, all pregnant women were universally recommended to undergo screening with a 50-g GCT irrespective of fasting at 24–28 GW and subsequent a 3-h 100-g oral glucose tolerance test (OGTT) with measurements of fasting insulin and HbA1c after more than a 8-h fasting if the 50-g GCT result was ≥ 140 mg/dL. The diagnosis of GDM and NGT depended on the Carpenter-Coustan criteria. From the total group of 7820 subjects, 251 subjects were excluded due to pregnancy-induced hypertension before 24 GW (n = 25), no maternal weight record (n = 28), and no result of GCT (n = 198). Out of 7569 subjects screened with a 50-g GCT, 1186 women with glucose ≥ 140 mg/dL on the 50-g GCT underwent a 100-g OGTT whereas 47 did not. Of these, 552 had NGT, 250 had impaired glucose tolerance, and 384 had GDM. From the 6888 NGT, 167 delivered at other hospital. As a result, 6721 NGT subjects were included in the study (Supplementary Fig. 1).

### Fetal biometry

Among 6721 NGT subjects, 5097 had fetal biometry data measured on the same day of 50-g GCT at 24–28 GW. Gestational dating was confirmed in 87% of these women by fetal ultrasonography performed prior to 14 GW. Biparietal diameter (BPD), femur length (FL), and abdominal circumference (AC) were measured three times via ultrasonography (GE Healthcare, USA) by one of the experienced three sonographers, and the mean values were converted to each estimated gestational age (GA) (i.e., GA-BPD, GA-FL, and GA-AC) according to the Japanese fetal growth chart^23,24^. But inter-observer variability was not evaluated due to the retrospective nature of this study. We calculated a set of fetal abdominal overgrowth ratios (FAORs) as GA-AC/GA-LMP (LMP, last menstruation period; actual GA measured by the LMP) to correct for the variations in the ultrasound scan timing, and GA-AC/GA-BPD or GA-AC/GA-FL to detect overgrowth of the abdomen relative to the head and femur growth, respectively. The presence of FAO was defined as FAORs ≥ 90th percentile of the total subjects with fetal biometry (GA-AC/GA-LMP ≥ 1.080, GA-AC/GA-BPD ≥ 1.071, and GA-AC/GA-FL ≥ 1.069, respectively). The estimated fetal weight was calculated using the Shinozuka formula^25^. We defined LGA at birth as ≥ 90th percentile of GA matched birth weight according to the report of Committee of the Korean Society of Neonatology by Lee et al.^26^. Macrosomia was defined as infant birth weight ≥ 4 kg.

### Biochemical analysis

Plasma glucose was measured using the hexokinase method (Quailigentglu, Sekisui, Japan), and HbA1c was measured via high-performance liquid chromatography (G8 Elution Buffer, Tosoh, Tokyo, Japan). The plasma insulin concentration was determined via electrochemiluminescenceimmunoassay (ElecsysInsulin, Roche Diagnostics GmbH, Mannheim, Germany). Insulin resistance (homeostatic model assessment for insulin resistance [HOMA-IR]) and secretion (HOMA-β) were calculated by homeostasis model assessment^27^.

### Subgroup analysis

For subgroup analysis, a total of 6721 NGT subjects were divided into four study groups according to maternal age and pre-pregnancy BMI—group 1 (age < 35 years and BMI < 25 kg/m^2^, n = 4226), group 2 (age < 35 years and BMI ≥ 25 kg/m^2^, n = 215), group 3 (age ≥ 35 years and BMI < 25 kg/m^2^, n = 2082), and group 4 (age ≥ 35 years and BMI ≥ 25 kg/m^2^, n = 158), and the NGT subgroup data were compared with each other.

For analysis according to the presence or absence of FAO, a total of 5097 subjects with fetal biometry data at 24–28 GW out of total 6721 NGT subjects were divided into NGT without FAO (n = 4637) and NGT with FAO (n = 492), and compared with each other.

### Statistical analyses

Clinical and biochemical characteristics of participants were summarized using mean with standard deviation (SD). Fetal biometry data and FAORs were described as mean and SD. Categorical and continuous pregnancy outcomes were summarized as mean with SD, and frequency and percentage, respectively. For continuous clinical and biochemical characteristics, fetal biometry, FAORs, and continuous pregnancy outcomes, two-sample *t-*test was used to compare total NGT and NGT by subgroups by maternal age and pre-pregnancy BMI pairwise. For categorical pregnancy outcomes, test of proportions was used to compare different groups of subjects pairwise. Logistic regression analysis models were used to estimate the odds of FAO in NGT group 2, 3, and 4 relative to that of NGT group 1. Another set of logistic regression analysis models was implemented to estimate the odds of LGA at birth, macrosomia, and primary cesarean delivery for the NGT group 2, 3, and 4 relative to group 1. Lastly, clinical characteristics and pregnancy outcomes were compared by the presence or absence of FAO at the time of diagnosis of GDM using two-sample *t-*test and test of proportions among NGT subjects. In analysis of supplementary data, correlations of FAORs with the clinical factors using Pearson’s correlation coefficient were assessed. Also, linear associations between each of FAORs and relevant clinical factors were estimated using multiple linear regression models with NGT subjects. The estimated odds of having FAO were analyzed with the consistent set of clinical factors using multiple logistic regression models. Finally, we examined the association between FAO and categories of maternal age (< 30, 30–35, 35–40, and > 40 years) and pre-pregnancy BMI (< 20, 20–25, 25–30, and > 30 kg/m^2^) adjusting for the relevant factors by fitting multiple logistic regression models. Same multivariable logistic regression models were analyzed for LGA at birth and macrosomia respectively as outcome as well. All analyses were conducted using STATA version 15.1 (Stata Corp LP, College Station, TX). The level of significance for the analyses was 0.05.

## Results

### Clinical and biochemical characteristics of NGT subgroups according to maternal age and BMI

Table 1 shows the clinical and biochemical characteristics of the NGT subgroups according to maternal age and pre-pregnancy BMI. Weight gain during pregnancy was significantly higher in group 1 than group 2, and 4. The glucose level on 50-g GCT in group 2 and 3 was significantly higher than group 1, but there were no significant differences in the fasting glucose on 100-g OGTT and HbA1c levels between the study groups. While HOMR-IR tended to be higher in group 2 without statistical significance, HOMA-β in group 3 was significantly lower than group 2.

**Table 1:** Clinical and biochemical characteristics of NGT and subgroups of NGT subjects according to maternal age and pre-pregnancy BMI.

	Total NGT	NGT			
		Group 1	Group 2	Group 3	Group 4
Number	6721	4226	215	2082	158
Clinical					
Age (years)	33.1 ± 3.8	31.0 ± 2.4^c,d^	31.2 ± 2.4^c,d^	37.2 ± 2.2^a,b^	37.5 ± 2.3^a,b^
Pre-pregnancy BMI (kg/m^2^)	20.6 ± 2.6	20.0 ± 1.9^b,d^	27.5 ± 2.6^a,^^c^	20.5 ± 1.9^b,d^	27.4 ± 2.4^a,^^c^
Weight gain (kg)					
Pre-pregnancy—at diagnosis	7.6 ± 3.2	7.7 ± 3.1^b,d^	5.8 ± 4.9^a^^,c^	7.6 ± 3.2^b,d^	5.3 ± 4.2^a,c^
Pre-pregnancy—near term	13.0 ± 4.1	13.3 ± 4.0^b,c,d^	11.0 ± 5.9^a^^,c^	12.7 ± 3.9^a,b^^,d^	10.1 ± 5.0^a,c^
Biochemical					
Glucose on 50-g GCT (mg/dL)	111.7 ± 21.1	109.9 ± 21.0^b,c^	116.2 ± 19.6^a^	114.3 ± 20.9^a^	118.9 ± 23.1
Fasting glucose on 100-g OGTT(mg/dL)	80.2 ± 6.4	80.4 ± 6.2	81.9 ± 6.8	80.3 ± 6.7	80.8 ± 6.1
HbA1c at diagnosis (%)	5.0 ± 0.3	5.0 ± 0.2	5.2 ± 0.4	5.0 ± 0.4	5.1 ± 0.2
HOMA-IR	1.61 ± 0.89	1.58 ± 086	3.21 ± 1.66	1.41 ± 0.60	1.76 ± 0.64
HOMA-β	181.0 ± 107.6	184.7 ± 111.1	359.9 ± 80.4^c^	155.4 ± 86.0^b^	144.8 ± 77.4

*NGT* normal glucose tolerance, *BMI* body mass index, *GCT* glucose challenge test, *OGTT* oral glucose tolerance test, *HbA1c* glycated hemoglobin, *HOMA-IR* homeostatic model assessment for insulin resistance, *HOMA-β* homeostatic model assessment for insulin secretion.

^a^*p* < 0.05 with Bonferroni correction for pairwise comparison, compared with NGT group 1; ^b^*p* < 0.05 with Bonferroni correction for pairwise comparison, compared with NGT group 2; ^c^*p* < 0.05 with Bonferroni correction for pairwise comparison, compared with NGT group 3; ^d^*p* < 0.05 with Bonferroni correction for pairwise comparison, compared with NGT group 4.

### Fetal biometry and FAORs of the NGT subgroups according to maternal age and BMI

Table 2 demonstrates the fetal biometry data and FAORs of the NGT subgroups according to maternal age and pre-pregnancy BMI at 24–28 GW measured simultaneously with 50-g GCT. Actual gestational age by LMP at fetal biometry performed as well as estimated GA of BPD and FL were not significantly different among the study groups. But the fetal biometry data of GA-AC and estimated fetal weight (EFW) in group 3 were significantly higher than group 1. The FAORs of GA-AC/GA-LMP and GA-AC/GA-BPD in group 3 and 4, and GA-AC/GA-FL in group 3 were significantly higher than group 1.

**Table 2:** Results of fetal biometry and FAORs measured at 50-g GCT, 24–28 gestational weeks in NGT and subgroups of NGT subjects.

	Total NGT	NGT			
		Group 1	Group 2	Group 3	Group 4
Number	5097	3252	165	1556	124
Fetal biometry					
GA-LMP^e^	26.4 ± 1.0	26.4 ± 1.0	26.3 ± 1.0	26.4 ± 0.9	26.4 ± 1.1
GA-AC (week)	27.1 ± 1.4	27.1 ± 1.4^b^	27.3 ± 1.5	27.2 ± 1.4^a^	27.3 ± 1.7
GA-BPD (week)	26.9 ± 1.5	26.9 ± 1.6	26.9 ± 1.6	26.9 ± 1.5	26.8 ± 1.6
GA-FL (week)	26.9 ± 1.4	26.9 ± 1.4	27.0 ± 1.4	26.9 ± 1.4	26.9 ± 1.6
EFW (gm)	1019.8 ± 162.9	1015.8 ± 164.4^b^	1017.3 ± 169.4	1028.2 ± 155.3^a^	1037.0 ± 190.0
FAOR					
GA-AC/GA-LMP^d^	1.028 ± 0.040	1.026 ± 0.039^b,c^	1.034 ± 0.043	1.033 ± 0.041^a^	1.04 ± 0.043
GA-AC/GA-BPD	1.010 ± 0.047	1.007 ± 0.046^b,c^	1.016 ± 0.044	1.013 ± 0.047^a^	1.02 ± 0.05^a^
GA-AC/GA-FL	1.010 ± 0.045	1.008 ± 0.044^b^	1.012 ± 0.050	1.014 ± 0.046^a^	1.02 ± 0.05

*FAOR* fetal abdominal overgrowth ratio, *GCT* glucose challenge test, *NGT* normal glucose tolerance, *GA-LMP* gestational age by last menstruation period, (n = 6921), *GA-AC* estimated gestational age by abdominal circumference, *GA-BPD* estimated gestational age by biparietal diameter, *GA-FL* estimated gestational age by femur length, *EFW* estimated fetal weight.

^a^*p* < 0.05 with Bonferroni correction for pairwise comparison, compared with NGT group 1; ^b^*p* < 0.05 with Bonferroni correction for pairwise comparison, compared with NGT group 3; ^c^*p* < 0.05 with Bonferroni correction for pairwise comparison, compared with NGT group 4; ^d^Gestational age by LMP at 50-g GCT.

### Prevalence of FAO and pregnancy outcomes of the NGT subgroups according to maternal age and BMI

Table 3 illustrates the prevalence of FAO at 24–28 GW and pregnancy outcomes of the NGT subgroups. The prevalence of FAO in group 3 was significantly higher than group 1 (11.8% vs. 8.6%, *p* < 0.01). For pregnancy outcomes, while GA at delivery in group 2, 3 and 4 was significantly lower than group 1, the rate of primary cesarean delivery in group 3 was significantly higher than group 1 (24.7% vs.19.8%, *p* < 0.001). Also, infant birth weight in group 2 and 4 and the rate of LGA in group 2, 3, and 4 (12.4%, 6.3%, and 11.5% vs. 4.4%, *p* < 0.001), and macrosomia in group 2 (5.0% vs. 1.8%, *p* < 0.05) were significantly higher than group 1.

**Table 3:** Pregnancy outcomes of the NGT and subgroups of NGT subjects according to maternal age and pre-pregnancy BMI.

	Total NGT	NGT			
		Group 1	Group 2	Group 3	Group 4
Number	6721	4226	215	2082	158
FAO at diagnosis^e^ (%)	9.6	8.6^c^	10.9	11.8^a^	8.9
Pregnancy outcomes					
Primipara (%)	4718 (70.2)	3279 (77.6)^b,c,d^	144 (67.0)^a,^^c,d^	1215 (58.4)^a,b^	79 (50.4)^a,b^
Cesarean delivery (%)	2426 (36.1)	1272 (30.1)^c,d^	96 (45.0)	959 (46.1)^a^	81 (51.7)^a^
Primary cesarean delivery (%)	1512 (22.5)	883 (20.9)^c^	55 (25.6)	535 (25.7)^a^	39 (24.9)
Infant birth weight (g)	3197 ± 421	3195 ± 406^b,d^	3307 ± 462^a^^,c^	3186 ± 437^b,d^	3286 ± 464^a,c^
GA at delivery (weeks)	39.0 ± 1.5	39.1 ± 1.4^b,c,d^	38.8 ± 1.4^a^	38.8 ± 1.5^a^	38.4 ± 1.9^a^
LGA (%)	342 (5.1)	169 (4.0)^b,c,d^	26 (12.4)^a,^^c^	131 (6.3)^a,b^^,d^	18 (11.5)^a,^^c^
Macrosomia^f^ (%)	127 (1.9)	76 (1.8)	10 (5.0)^a^	39 (1.9)^b^	3 (2.2)

*NGT* normal glucose tolerance, *BMI* body mass index, *FAO* fetal abdominal obesity, *GA* gestational age, *LGA* large for gestational age.

^a^*p* ≤ 0.05 with Bonferroni correction for pairwise comparison, compared with NGT group 1; ^b^*p* ≤ 0.05 with Bonferroni correction for pairwise comparison, compared with NGT group 2; ^c^*p* ≤ 0.05 with Bonferroni correction for pairwise comparison, compared with NGT group 3; ^d^*p* < 0.05 with Bonferroni correction for pairwise comparison, compared with NGT group 4; ^e^N = 5097; ^f^Infant birth weight ≥ 4 kg.

### Odds ratio for FAO of the NGT subgroups according to maternal age and BMI

FAO, defined as FAOR ≥ 90%, was GA-AC/GA-LMP ≥ 1.080, GA-AC/GA-BPD ≥ 1.071, and GA-AC/GA-FL ≥ 1.069, respectively, Relative to group 1, the adjusted odds ratio for FAO by GA-AC/GA-LMP in group 3 was 1.42 (95% confidence interval (CI) 1.17–1.73), by GA-AC/GA-BPD in group 4 was 1.84 (95% CI 1.11–3.05) and by GA-AC/GA-FL in group 3 and 4 was 1.31 (95% CI 1.07–1.60) and 1.90 (95% CI 1.15–3.15), respectively (Table 4).

**Table 4:** Odds ratio for fetal abdominal obesity (FAO) in subgroups of NGT subgroups.

	Odds ratio (95% CI) FAO^a^		
	GA-AC/GA-LMP^b^ ≥ 90th	GA-AC/GA-BPD ≥ 90th	GA-AC/GA-FL ≥ 90th
NGT Group 1	1 (ref.)	1 (ref.)	1 (ref.)
NGT Group 2	1.31 (0.79, 2.16)	1.09 (0.64, 1.85)	1.37 (0.84, 2.24)
NGT Group 3	1.42 (1.17, 1.73)^c^	1.17 (0.96, 1.44)	1.31 (1.07, 1.60)^c^
NGT Group 4	1.04 (0.55, 1.95)	1.84 (1.11, 3.05)^c^	1.90 (1.15, 3.15)^c^

*GA-AC* estimated gestational age by abdominal circumference, *GA-LMP* gestational age by last menstruation period, *GA-BPD* estimated gestational age by biparietal diameter, *GA-FL* estimated gestational age by femur length, *NGT* normal glucose tolerance.

^a^Fetal abdominal obesity, defined as fetal abdominal overgrowth ratio (FAOR) ≥ 90th percentile, is GA-AC/GA-LMP ≥ 1.080, GA-AC/GA-BPD ≥ 1.071, and GA-AC/GA-FL ≥ 1.069; ^b^Gestational age by LMP; ^c^*p* < 0.05.

### Odds ratios for LGA at birth, macrosomia, and primary cesarean delivery of the NGT subgroups according to maternal age and BMI

Relative to group 1, the adjusted odds ratio for LGA at birth was 3.06 (95% CI 1.96–4.77, *p* <0.005), 1.47 (1.16–1.86, *p* <0.005) and 2.82 (1.64–4.84, *p* <0.005) in group 2, 3 and 4, respectively, for macrosomia was 2.53 (1.29–4.96, *p* <0.05) in group 2, and for primary cesarean delivery in group 3 was 1.33 (1.18–1.51, *p* <0.005) (Table 5).

**Table 5:** Odds ratio for LGA at birth, macrosomia, and primary cesarean delivery in NGT subgroups.

	Odds ratio (95% CI) Pregnancy outcomes		
	LGA at birth	Macrosomia	Primary cesarean delivery
NGT Group 1	1 (ref.)	1 (ref.)	1 (ref.)
NGT Group 2	3.06 (1.96, 4.77)^a^	2.53 (1.29, 4.96)^b^	1.32 (0.97, 1.80)
NGT Group 3	1.47 (1.16, 1.86)^a^	0.94 (0.64, 1.40)	1.33 (1.18, 1.51)^a^
NGT Group 4	2.82 (1.64, 4.84)^a^	1.07 (0.33, 3.43)	1.28 (0.88, 1.84)

*LGA* large for gestational age, *NGT* normal glucose tolerance.

^a^*p* < 0.005; ^b^*p* < 0.05.

### Clinical characteristics and pregnancy outcomes according to presence of FAO in the total NGT subjects

The NGT subjects with FAO observed at 24–28 GW were significantly older, more obese, and gained more weight from pre-pregnancy to 24–28 GW compared with those without FAO (Table 6). Plasma glucose level on 50-g GCT, fasting glucose on 100-g OGTT, HOMA-IR, and HOMA-β were not differ between the two groups. Significantly higher prevalence of FAO near term, male sex, LGA at birth and macrosomia, and the tendency to higher cesarean delivery were observed in the NGT subjects with FAO at 24–28 GW than those without FAO.

**Table 6:** Clinical characteristics and pregnancy outcomes depending on the presence or absence of FAO at 50-g GCT, 24–28 GW in the subjects with NGT.

	Total NGT (N = 5097)		
	FAO ( −) (n = 4608)	FAO ( +) (n = 489)	*p* Value
Clinical			
Age (years)	33.0 ± 3.7	33.7 ± 3.7	< 0.001
Pre-pregnancy BMI (kg/m^2^)	20.5 ± 2.6	21.0 ± 2.6	< 0.001
Weight change (kg)			
Pre-pregnancy—at diagnosis	7.5 ± 3.3	8.1 ± 3.3	< 0.001
Pre-pregnancy—near term	13.0 ± 4.1	13.3 ± 4.0	0.1445
HbA1c at diagnosis (%)*	5.00 ± 0.26	5.05 ± 0.20	0.2560
Glucose on 50-g GCT (mg/dL)	111.5 ± 21.2	113.4 ± 21.0	0.0698
Fasting glucose on 100-g OGTT (mg/dL)*	80.0 ± 6.2	80.8 ± 6.2	0.4090
HOMA-IR*	1.67 ± 0.91	1.32 ± 0.69	0.3535
HOMA-β*	190.37 ± 117.15	161.21 ± 46.67	0.8410
FAO (+) near term (%)	7.8	27.1	< 0.001
Pregnancy outcomes			
Primipara (%)	67.9	67.1	0.7169
Male sex of infant (%)	50.7	64.4	< 0.001
LGA at birth (%)	4.1	16.2	< 0.001
Macrosomia (%)	1.5	6.4	< 0.001
Primary cesarean delivery (%)	21.0	26.4	0.0050

*FAO* fetal abdominal obesity, *GCT* glucose challenge test, *GW* gestational week, *NGT* normal glucose tolerance, *BMI* body mass index, *HbA1c* glycated hemoglobin, *HOMA-IR* homeostatic model assessment for insulin resistance, *HOMA-β* homeostatic model assessment for insulin secretion, *LGA* large for gestational age.

*Significantly smaller number of observations were available; n = 411 for FPG on 100-g OGTT, n = 363 for HbA1c at diagnosis, and n = 59 for HOMA-IR and HOMA-β.

### Correlation of FAORs with clinical factors

FAORs showed significant positive correlation with maternal age, pre-pregnancy BMI, BMI at diagnosis, weight gain until diagnosis, plasma glucose on 50-g GCT (Supplementary Table 1).

### Multiple linear regression analysis of FAORs with clinical factors

Pre-pregnancy BMI, weight gain until diagnosis of GDM, maternal age, and plasma glucose on 50-g GCT showed linear relationship with FAOR of GA-AC/GA-LMP using multiple linear regression models among NGT subjects (Supplementary Table 2).

### Multiple logistic regression analysis of FAO with clinical factors

Maternal age, pre-pregnancy BMI, and weight gain until 24–28 GW were independent predictor for FAO by multiple logistic regression analysis (Supplementary Table 3).

### Odds ratios for FAO, LGA at birth, and macrosomia in the NGT subjects categorized by maternal age and pre-pregnancy BMI

After adjusting pre-pregnancy BMI, weight gain until diagnosis, and glucose level on 50-g GCT, maternal age of 35–40 years showed odds ratio for FAO 1.55 (95% CI 1.13–2.11, *p* < 0.05), relative to age < 30 years. After adjusting maternal age and other factors, odds ratio for FAO of pre-pregnancy BMI 20–25 kg/m^2^ was 1.41 (1.16–1.72, *p* < 0.05) (Supplementary Table 4).

Relative to pre-pregnancy BMI < 20 kg/m^2^, adjusted odds for LGA at birth of pre-pregnancy BMI 20–25 kg/m^2^, 25–30 kg/m^2^, and > 30 kg/m^2^ were 2.11 (1.64–2.72, *p* < 0.05), 4.21 (2.75–6.45, *p* < 0.05), and 9.38 (4.12–21.33, *p* < 0.05) (Supplementary Table 5).

Relative to pre-pregnancy BMI < 20 kg/m^2^, adjusted odds for macrosomia of pre-pregnancy BMI 20–25 kg/m^2^, 25–30 kg/m^2^, and > 30 kg/m^2^ were 2.51 (1.67–3.78, *p* < 0.05), 4.09 (2.03–8.23, *p* < 0.05), and 6.07 (1.38–26.62, *p* < 0.05) (Supplementary Table 6).

## Discussion

Prevalence of advanced maternal age and obesity in women of reproductive age is increasing^28–32^. Advanced maternal age, defined as childbearing over 35 years of age^33^, have emerged as an increasingly important issue due to its association with comorbidities^28–30^ and many adverse pregnancy outcomes such as PIH, premature delivery, fetal growth restriction, and postpartum hemorrhage^13,14,34–38^. Furthermore maternal obesity is well known to increases the risk of fetal overgrowth resulting macrosomia, large for gestational age, greater amount of fat mass^7,16^, and higher BMI associated with later cardiovascular and metabolic risk profile^20^.

We previously observed the increased risk of FAO in the older women with or without obesity but not in the young and non-obese women with GDM^12,21,22^, but the prevalence of FAO in the NGT subjects were not elucidated yet. In the present study, we observed significantly increased FAO in the older women with or without obesity compared with the younger and non-obese women in the NGT subjects. Moreover, the FAO detected at 24–28 GW persisted until near term and resulted in adverse perinatal outcomes such as increased primary cesarean delivery, LGA and macrosomia. These findings suggest that even the NGT in the older women with or without obesity should be regarded as high-risk pregnancy, and appropriate intervention including the management of FAO would be necessary to improve the perinatal outcomes of this high-risk pregnancy population.

Measuring AC by fetal ultrasound can be used as a reliable marker of fetal adiposity^39^. We have assessed FAO with FAORs of ultrasonographically estimated GA of abdominal circumference per actual GA by LMP, BPD or FL, respectively. In our previous studies^12,21,22^, we observed that FAORs ≥ 90th percentile was more sensitive than AC ≥ 90th percentile for detecting FAO, since about 2 weeks’ acceleration of abdominal growth meets the FAORs criteria, but more than 3 weeks’ acceleration meets the AC criteria for FAO at 26 GW.

According to our current data that significantly higher FAORs of older group 3 and 4 and more frequent FAO in older/non-obese group 3 in comparison with younger/non-obese group, fetal abdominal growth was accelerated in older NGT groups. In addition, a significantly increased odds ratio for FAO compared to the younger/non-obese group 1 NGT subjects were observed only in the older/non-obese group 3 (1.31) and the older/obese group 4 (1.90), but not in the younger/obese group 2. This finding suggest that elderly pregnancy is the primary risk factor for FAO at 24–28 GW, and the co-presence of obesity more aggravates the FAO observed in the elderly older NGT pregnant women.

It is well known that age is an independent predictor of beta-cell function^40,41^. The findings of the lowest HOMA-β in group 3 and 4, and the slightly increased tendency of HOMA-IR in group 4 than group 1 NGT subjects suggest that decreased insulin secretion due to advancing maternal age is primarily responsible, and increased insulin resistance due to obesity might have additive effects, for the FAO observed in the older NGT women with or without obesity. Also, this finding was in concord with the Madsen et al.’s report that maternal insulin secretion and insulin sensitivity is superior to presence or absence of GDM in predicting neonatal adiposity and neonatal hyperinsulinemia^42^.

On the other hands, the odd ratio for LGA at birth was significantly higher as 3.06 and 2.82 in obese group 2 and 4 with reference to group1. The odds ratio was also significantly higher in older/non-obese group 3 as 1.47. The odds ratio for macrosomia was higher as 2.53 only in younger/obese group 2 but not in older/obese group 4. From these results, it can be said that maternal obesity mainly affects infant birth weight^15–17^ and maternal age modify the effect with placental dysfunction, as reported by others^43–46^.

Looking at the clinical characteristics and pregnancy outcomes of the subjects with FAO at the time of GDM screening, their maternal age and pre-pregnancy BMI were significantly higher than the subjects without FAO, while the difference between the actual ages (33.0 vs. 33.7 years) and pre-pregnancy BMI (20.5 vs. 21.0 kg/m^2^) of those groups were not so big. But LGA at birth and macrosomia frequency was fourfold higher in the subjects with FAO and prevalence of FAO near term was threefold higher in the group with FAO than in the group without FAO at GDM screening.

On the other hand, the prevalence of LGA at birth is significantly lower compared with the FAO near term and the fact that only 16.2% of fetuses with FAO at the time of GDM screening were born as LGA suggest that significant portion of infants with FAO in the NGT subjects have relative abdominal overgrowth but appropriate body weight for GA at birth. These findings are consistent with our previous and other studies^3,12,21,22^ showing that infants of women with GDM have increased body fat despite having an average weight for GA.

In multiple logistic regression analyses, increase in maternal age, pre-pregnancy BMI, glucose level on 50 g-GCT, and maternal weight gain until 24–28 GW were clinical factors significantly associated with FAO. These findings suggest that planned pregnancy along with preconception care to maintain appropriate BMI, normal glucose tolerance, and the best management for appropriate weight gain starting from early pregnancy would be necessary to prevent the FAO in the older NGT women with or without obesity.

The limitations of the present study include the single center, retrospective, and uncontrolled study design. Although the same ultrasound scanners were used, and all pregnant women scanned were randomly assigned to one of the three sonographers, we could not assess the inter-observer variability of the obtained fetal ultrasound data due to retrospective nature of this study. However, the strengths of this study include a relatively large sample size with the same ethnicity and clinical management of all subjects according to the same protocol during the study period.

In summary, while the prevalence of LGA at birth was significantly higher in obese women than non-obese women, the prevalence of FAO at 24–28 GW was significantly higher in the older NGT women with or without obesity compared with the younger/non-obese NGT subjects. Moreover, FAO detected at 24–28 GW persisted near term, and resulted in the adverse perinatal outcomes such as increased cesarean delivery, LGA and macrosomia. These findings suggest that active interventions including maintenance of normal BMI and body weight gain starting before or early in pregnancy would be necessary to prevent FAO ultimately leading to metabolic abnormalities even up to child- and adulthood, especially in the high risk older with/without obese NGT pregnant women.

### Supplementary Information

Supplementary Information.

## Data Availability

The study protocol and statistical code are available from the corresponding author, and data are available to approved individuals through written agreements with the authors and the data partner.
