# Supplementary material for: Fetal abdominal obesity and the ensuing adverse perinatal outcomes in older obese pregnant women with or without obesity and with normal glucose tolerance
Source: Sci Rep. 2023 Sep 27;13:16206. doi: 10.1038/s41598-023-43362-w (PMC10533511; doi:10.1038/s41598-023-43362-w)
Supplement: Supplementary file 1 — Supplementary Information. [file 41598_2023_43362_MOESM1_ESM.pdf]

Supplementary Table 1. Correlation of FAORs with clinical factors in NGT subjects

GA-AC/GA-LMP

Clinical factors	Correlation Coeff.	p-value
Age	0.0939	<0.0001
Pre-pregnancy BMI	0.0938	<0.0001
BMI at diagnosis	0.1249	<0.0001
Weight gain until diagnosis	0.1044	<0.0001
Glucose on 50-g GCT	0.0523	0.0002
Fasting glucose on 100-g OGTT	-0.0472	0.3397
HbA1c	0.0226	0.6677

GA-AC/GA-BPD

Clinical factors	Correlation Coeff.	p-value
Age	0.0782	<0.0001
Pre-pregnancy BMI	0.1027	<0.0001
BMI at diagnosis	0.0957	<0.0001
Weight gain until diagnosis	0.0081	0.5629
Glucose on 50-g GCT	0.0387	0.0058
Fasting glucose on 100-g OGTT	-0.0224	0.6530
HbA1c	0.0750	0.1566

GA-AC/GA-FL

Clinical factors	Correlation Coeff.	p-value
Age	0.0625	<0.0001
Pre-pregnancy BMI	0.0654	<0.0001
BMI at diagnosis	0.0904	<0.0001
Weight gain until diagnosis	0.0666	<0.0001
Glucose on 50-g GCT	0.0239	0.0881
Fasting glucose on 100-g OGTT	-0.0075	0.8789
HbA1c	0.0326	0.5353

GA-AC, estimated gestational age by abdominal circumference; GA-LMP, gestational age by last menstruation period; BMI, body mass index; GCT, glucose challenge test; OGTT, oral glucose tolerance test; HbA1c, glycated albumin; GA-BPD, estimated gestational age by biparietal diameter; GA-FL, estimated gestational age by femur length

Supplementary Table 2. Estimated coefficients of clinical factors showing linear relationship with FAORs using multiple linear regression models among NGT subjects

GA-AC/GA-LMP

Clinical factors	Coef.	Std. Err.	t	P-value	[95% Conf.	Interval]
Age	0.0008623	0.0001519	5.68	0.000	0.0005645	0.0011602
Pre-pregnancy BMI	0.0013804	0.0002222	6.21	0.000	0.0009449	0.0018159
Weight gain until diagnosis	0.0012506	0.0001718	7.28	0.000	0.0009137	0.0015875
Glucose on 50-g GCT	0.0000546	0.0000266	2.05	0.040	2.43E-06	0.0001068
_cons	0.9559987	0.0069104	138.34	0.000	0.9424513	0.9695462

GA-AC/GA-BPD

Clinical factors	Coef.	Std. Err.	t	P-value	[95% Conf.	Interval]
Age	0.0007949	0.0001774	4.48	0.000	0.0004471	0.0011427
Pre-pregnancy BMI	0.0014596	0.0002596	5.62	0.000	0.0009505	0.0019686
Weight gain until diagnosis	0.0002666	0.0002008	1.33	0.184	-0.000127	0.0006602
Glucose on 50-g GCT	0.0000421	0.0000312	1.35	0.177	-0.000019	0.0001032
_cons	0.9465159	0.0080857	117.06	0.000	0.9306645	0.9623673

GA-AC/GA-FL

Clinical factors	Coef.	Std. Err.	t	P-value	[95% Conf.	Interval]
Age	0.0006451	0.0001714	3.76	0.000	0.0003091	0.0009811
Pre-pregnancy BMI	0.0011226	0.0002505	4.48	0.000	0.0006314	0.0016138
Weight gain until diagnosis	0.0008336	0.000194	4.3	0.000	0.0004533	0.0012139
Glucose on 50-g GCT	0.0000389	0.00003	1.29	0.196	-0.00002	0.0000977
_cons	0.9545633	0.0077973	122.42	0.000	0.9392772	0.9698493

GA-AC, estimated gestational age by abdominal circumference; GA-LMP, gestational age by last menstruation period; BMI, body mass index; GCT, glucose challenge test; GA-BPD, estimated gestational age by biparietal diameter; GA-FL, estimated gestational age by femur length

Supplementary Table 3. Estimated odds ratios of clinical factors for FAO at diagnosis of GDM using multiple logistic regression model among NGT subjects

**Clinical factors**	**Odds Ratio**	**Std. Err.**	**z**	**P-value**	**[95% Conf.**	**Interval]**
Age	1.04441	0.0134545	3.37	0.001	1.01837	1.071116
Pre-pregnancy BMI	1.066942	0.0189468	3.65	0.000	1.030445	1.104731
Weight gain until diagnosis	1.06168	0.0153419	4.14	0.000	1.032032	1.092179
Glucose on 50-g GCT	1.002198	0.0022814	0.96	0.335	0.9977368	1.00668
_cons	0.0031912	0.0018762	-9.78	0.000	0.0010081	0.0101019

FAO, fetal abdominal obesity; GDM, gestational diabetes mellitus; NGT, normal glucose tolerance;

BMI, body mass index; GCT, glucose challenge test

Supplementary Table 4. Estimated odds ratios for FAO in subjects categorized by maternal age and pre-pregnancy BMI with adjustment of other factors.

**Variable**	**FAO** Odds ratio (95% CI)			
**Maternal Age** [a] **(years)**	**< 30 (ref.)**	**30-35**	**35-40**	**> 40**
	1.00	1.20 (0.89, 1.62)	1.55 (1.13, 2.11)	1.56 (0.99, 2.46)
**Pre-pregnancy**[b] **BMI (kg/m^2)**	**< 20 (ref.)**	**20-25**	**25-30**	**> 30**
	1.00	1.41 (1.16, 1.72)	1.24 (0.79, 1.94)	2.15 (0.82, 5.66)

FAO, fetal abdominal obesity; BMI, body mass index

[a] adjusted for pre-pregnancy BMI category, weight gain until diagnosis, and glucose on 50-g GCT;

[b] adjusted for maternal age group, weight gain until diagnosis, and glucose on 50-g GCT, $p < 0.005$

Supplementary Table 5. Estimated odds ratios for LGA at birth in subjects categorized by maternal age and pre-pregnancy BMI with adjustment of other factors

Variable	LGA Odds ratio (95% CI)			
**Maternal Age [a] (years)**	**< 30 (ref.)**	**30-35**	**35-40**	**> 40**
	1.00	0.99 (0.70, 1.41)	1.36 (0.95, 1.96)	1.14 (0.66, 1.97)
**Pre-pregnancy[b] BMI (kg/m^2)**	**< 20 (ref.)**	**20-25**	**25-30**	**> 30**
	1.00	2.11 (1.64, 2.72)	4.21 (2.75, 6.45)	9.38 (4.12, 21.33)

LGA, large for gestational age; BMI, body mass index

[a] adjusted for pre-pregnancy BMI category, weight gain diagnosis, and glucose on 50-g GCT;

[b]adjusted for maternal age group, weight gain until diagnosis, and glucose on 50-g GCT, $p < 0.005$

Supplementary Table 6. Estimated odds ratios for macrosomia at birth in subjects categorized by maternal age and pre-pregnancy BMI with adjustment of other factors

**Variable**	**Macrosomia** Odds ratio (95% CI)			
**Maternal Age[a] (years)**	**< 30 (ref.)**	**30-35**	**35-40**	**> 40**
	1.00	0.72 (0.45, 1.15)	0.69 (0.41, 1.17)	0.47 (0.18,1.24)
**Pre-pregnancy[b] BMI (kg/m^2)**	**< 20 (ref.)**	**20-25**	**25-30**	**> 30**
	1.00	2.51 (1.67, 3.78)	4.09 (2.03, 8.23)	6.07 (1.38, 26.62)

[a] adjusted for pre-pregnancy BMI category, weight gain diagnosis, and glucose on 50-g GCT; [b]adjusted for maternal age group, weight gain until diagnosis, and glucose on 50-g GCT, p <0.005

Supplementary figure 1. Flow chart of study population

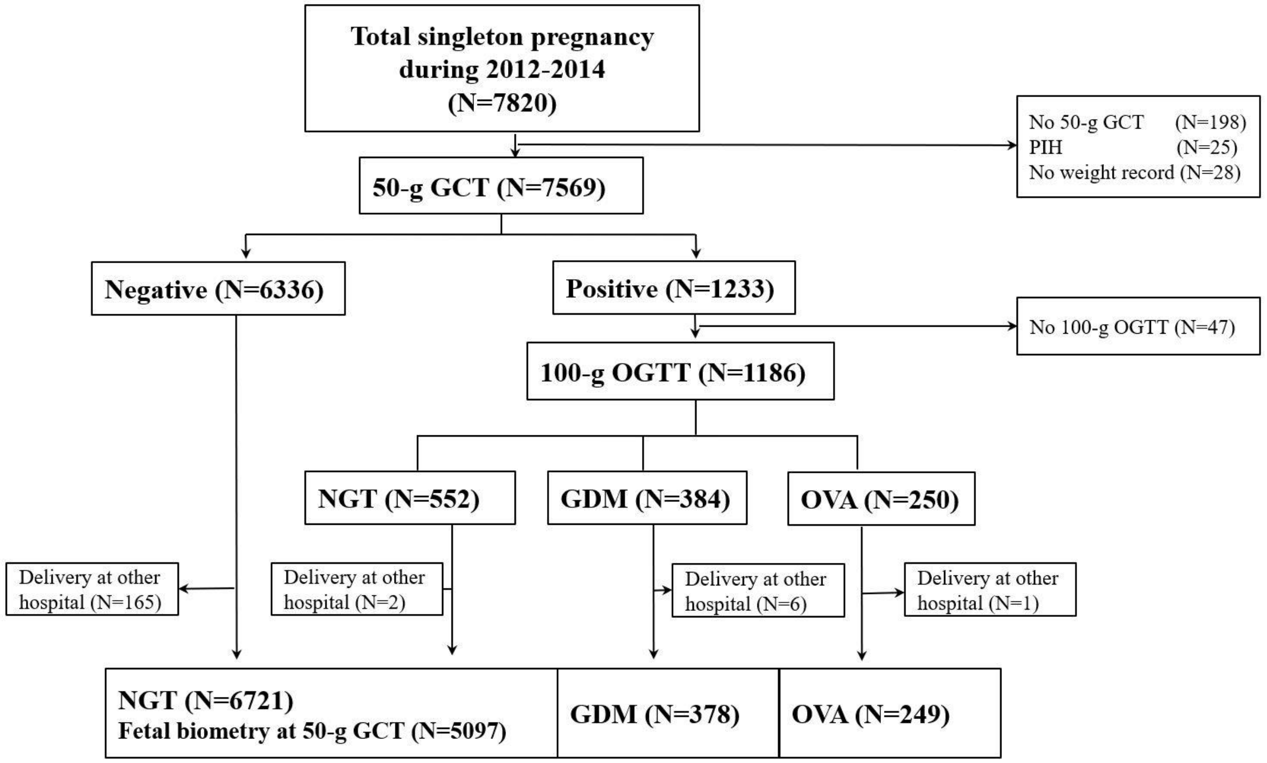

GCT, glucose challenge test; PIH, pregnancy induced hypertension; OGTT, oral glucose tolerance test; NGT, normal glucose tolerance; GDM, gestational diabetes mellitus; OVA, one value abnormality
